# Establishment and evaluation of a rapid method for the detection of bacterial pneumonia in hospitalized patients via multiplex PCR–capillary electrophoresis (MPCE)

**DOI:** 10.1128/spectrum.01202-24

**Published:** 2024-09-18

**Authors:** Jie Wang, Pei Zhao, Mengchuan Zhao, Duoxiao Zhang, Shan Chen, Ying Liu, Yuan Gao, Yanqing Tie, Zhishan Feng

**Affiliations:** 1 Department of Laboratory Diagnosis, Hebei Medical University, Shijiazhuang, Hebei, China; 2 Department of Clinical Laboratory, Hebei General Hospital, Shijiazhuang, Hebei, China; 3 Hebei Key Laboratory of Molecular Medicine, Hebei Clinical Research Center for Laboratory Medicine, Shijiazhuang, Hebei, China; 4 Department of Reagent Research and Development, Ningbo Health Gene Technologies Co., Ltd, Ningbo, Zhejiang, China; MultiCare Health System, Tacoma, Washington, USA

**Keywords:** bacteria, pneumonia, multiplex PCR, capillary electrophoresis

## Abstract

Cost-effective molecular diagnostic techniques for bacterial pneumonia are limited. We designed primers for 13 bacteria, performed multiplex nucleic acid detection through fragment analysis to obtain pathogen identification results, and established a multiplex PCR–capillary electrophoresis (MPCE) method, which can simultaneously detect 13 pathogens associated with bacterial pneumonia. The sensitivity, specificity, and reproducibility of the MPCE assay were tested, and 420 clinical samples were used to assess the clinical detection ability of MPCE, with the culture method used as a reference. Samples with inconsistent results detected by the two methods were sent for Sanger sequencing. The minimum detection limit of MPCE for 13 bacteria was 6.0 × 10^3^ cfu/mL~2.0 × 10^6^ cfu/mL. No cross-reactivity was observed with other pathogens. The percentage of agreement for reproducibility analysis reached 100%. For the 420 sputum samples, when the culture method was used as the reference, the sensitivity of MPCE to 13 bacteria ranged from 80% to 100%. The specificity for 13 bacteria ranged from 67.1% to 100%. The percentage of agreement between the MPCE and the culture method ranged from 69.7% to 100%. There was no statistically significant difference (*P* > 0.05) in the detection of *Escherichia coli*, *Enterobacter cloacae complex*, *Staphylococcus aureus*, methicillin-resistant *S. aureus, Streptococcus pyogenes*, *Moraxella catarrhalis*, or *Legionella pneumophila* between the MPCE and the culture method. Clinical samples with negative cultures but positive MPCE results were validated by Sanger sequencing, and the results were consistent with those of MPCE. The MPCE method has high sensitivity and specificity for bacterial pneumonia, enabling the simultaneous and rapid detection of multiple pathogens. It is cost-effective and has potential for clinical application.

**IMPORTANCE:**

This study successfully established a multiplex PCR–capillary electrophoresis detection system that can simultaneously detect 13 pathogens through a single detection method, significantly improving clinical efficiency. It is cost-effective and has potential for clinical application.

## INTRODUCTION

Pneumonia is a common infectious disease that is the primary reason that hospitals use antibiotics (1). Despite a better understanding of the etiology, pathogenesis, risk factors, and preventive care of pneumonia, it remains a significant cause of morbidity and mortality worldwide (2, 3). Especially in intensive care units (ICUs), the diagnosis of nosocomial pneumonia is associated with a worsening prognosis, with mortality rates ranging from 12% to 48% (4). Bacteria are major pathogens that cause nosocomial pneumonia, including *Klebsiella pneumoniae*, *Acinetobacter baumannii*, *Pseudomonas aeruginosa*, *Enterobacter cloacae complex*, *Stenotrophomonas maltophilia*, *Escherichia coli*, *Staphylococcus aureus*, methicillin-resistant *Staphylococcus aureus*, *Streptococcus pneumoniae*, *Streptococcus pyogenes*, *Legionella pneumophila*, *Haemophilus influenzae*, and *Moraxella catarrhalis* (1, 5–7). These bacterial infections or secondary infections often exacerbate symptoms of respiratory diseases. If the pathogen species cannot be identified in a timely manner and targeted medication is not administered, the optimal treatment time will be delayed, which may also cause an epidemic of nosocomial infection. Therefore, rapid and accurate identification of pathogens causing bacterial pneumonia is crucial for early diagnosis, treatment, and nosocomial infection prevention and control.

At present, pathogenic examination techniques commonly used in clinical practice include serology, microscopy, culture, and traditional molecular methods. However, routine serological indicators cannot accurately identify the pathogenic bacteria causing pneumonia, and there is significant blindness in the use of advanced antibiotics. Inappropriate empirical antibiotic treatment is common and is associated with increased mortality in critically ill patients (5). The classic bacterial culture method has long detection cycles and low sensitivity, is easily affected by antibiotics and other drugs, and cannot easily detect mixed infections (1). The genotype identification method using molecular technology has high sensitivity. The commercially available reagents used in clinical practice mainly cover common viruses and atypical pathogens such as *Mycoplasma pneumoniae*. There are areas for improvement in the detection of bacteria, such as inconsistent coverage of species with local epidemic strains (7) or high reagent costs. Our investigation revealed that the commercial reagents currently used in China for detecting bacterial pneumonia mainly use multiplex fluorescence quantitative PCR and isothermal amplification chip methods. The target coverage of multiplex fluorescence PCR is insufficient, and the detection range is small. The isothermal amplification chip method has a high cost and low flux. The cost of metagenomic next-generation sequencing is high. Few testing kits can simultaneously detect and distinguish the 13 pathogens that cause bacterial pneumonia while also being cost-effective.

In this study, we designed specific primers for 13 major bacteria that cause bacterial pneumonia and established a multiplex PCR–capillary electrophoresis (MPCE) method. Single-tube multiplex amplification was performed on the extracted and purified nucleic acid, and multiplex nucleic acid detection was performed through fragment analysis. Thirteen bacteria could be detected simultaneously, and the sensitivity and specificity of the detection method were evaluated.

## MATERIALS AND METHODS

### Clinical samples

Lower respiratory tract sputum samples were collected from patients diagnosed with pneumonia or pulmonary infection by doctors at Hebei General Hospital from May 2023 to December 2023 (8). The inclusion criteria were as follows: a sputum volume ≥0.6 mL; the sputum smear was subjected to Gram staining, and the quality was sufficient, with white blood cells >25/LP and epithelial cells <10/LP. The exclusion criteria were as follows: saliva or nasopharyngeal secretions; the quality of the sputum smear was found to be insufficient, with white blood cells <25/LP or epithelial cells >10/LP; and repeated specimens from the same patient.

### Nucleic acid extraction

An equal volume of physiological saline was added to the sputum sample, and the sample was thoroughly shaken via a vortex shaker for 2 min. After settling for 1–2 min, 300 µL of sputum supernatant was collected for nucleic acid extraction via an A-96 fully automatic nucleic acid extractor and a matching nucleic acid extraction or purification kit (magnetic bead method) (Health Gene, Ningbo) according to the manufacturer’s instructions. One hundred microliters of positive control (recombinant plasmid containing all tested pathogens and human DNA) (Haiyi Gene Technology Co., Ningbo), 100 µL of negative control (recombinant plasmid containing human DNA), and 2 µL of internal control (recombinant plasmid containing an internal control gene) were added to extract nucleic acid together. After extraction, the purity of the nucleic acid was estimated by the A260/A280 ratio via an ultramicrospectrophotometer. The ratio needed to be greater than 1.8 for each sample extract to be acceptable. The extracts were stored at −20°C until assayed.

### Primer design

Primers were designed on the basis of the reference sequence (Table 1) of 13 bacterial genes and human DNA via Oligo7.6 with the following parameters: (i) search strictness: very high; (ii) monovalent ion concentration: 50 mM; (iii) free Mg^2+^ concentration: 3 mM; (iv) equivalent total Na^+^ concentration: 269.1 mM; (v) primer length: 20–30 nt; (vi) primer Tm range: 60°C ± 1°C; and (vii) PCR product length range: 80–400 bp. The designed primers should be compared via BLAST and primer-BLAST to confirm their conservation and specificity. All primers were synthesized by Thermo Fisher Scientific (Shanghai, China). Each pair of primers was modified by labeling the 5′ end of the upstream or downstream primer with the fluorescent dye fluorescein phosphoramidite (FAM). To separate the products by electrophoresis and avoid the impact of peak-dragging after the saturation of each target on the interpretation of previous targets, the size difference between adjacent PCR products was approximately 10 bp.

**TABLE 1:** Primer sequences used in multiplex PCR^*a*^

Targets	Gene	NCBI ID of reference sequence	Genome position	Primer sequence (5′–3′) Primer-F Primer-R	Amplification product size (bp)	Final concentration (nM)
MRSA	*mecA*	NG_047937.1	1,643–1,752	ttGCTGATTCAGGTTACGGACAAG	113	250
				gTTTAATAAGTGAGGTGCGTTAATATTGC		250
PA	PA1767	NC_002516.2	1,912,080–1,912,195	ttcTGCATCTGAAAGTCCTGATCG	123	150
				gtatGTTCCAGAGGTCATCGGTTT		150
MC	*lepA*	NZ_CP018059.1	1,924,022–1,924,147	ttaaGTTTGTGGTGGTTTGTCAGACC	134	150
				gtaaGCCATTGGGATGATTATAGTACA		150
SPY	*speB*	NZ_LS483338.1	1,607,066–1,607,205	ttTCGGTAAAGTAGGCGGACAT	145	200
				gtaACCAGTACCAAGAGCTGAAG		200
SPN	SPNHU17_RS05160	NZ_CP020549.1	1,023,935–1,024,092	tTGTTGCTCTCTTAACGATGAAG	160	150
				gTTCATTTGTTCtACATTGTTGAAGTTT		150
HI	*fucA*	NZ_CP007470.1	1,031,880–1,032,047	ttACGTTAGTGTTCGTTATAAAGATGGAAT	173	150
				gttGGCGAGTATGATAAACACTCAAG		150
KP	KPHS_16050	NC_016845.1	1,666,779–1,666,955	taaAGGAATTTCTGTACGTTGGC	183	150
				taaCACAGCCCTGATTGTAGGAG		150
SA	SAOUHSC_01837	NC_007795.1	1,742,692–1,742,898	cCGTCTTTAAATGCATTTCCTGTAGAT	210	150
				gtGACCAACATAAGCAACAGGTAATA		150
SM	DQN92_RS09665	NZ_LS483377.1	3,870,749–3,870,974	ttcATCCTCAACATCGTCGAGTG	232	150
				gttGGTACACGATCGACAGCATC		150
LP	*wipC*	NZ_CP015941.1	2,454,337–2,454,559	ctaatCGCCAATCGATTTAGGAATAATCT	233	200
				gtattTCACGATTCCACAATATTTGCT		200
ECO	*oxc*	NC_002695.2	3,218,822–3,219,064	tctcAGAACCAGGATATTTATTTAGTTAATGA	250	150
				gttGCAGGTTATATCGACAAATCGTTTC		150
AB	F3P16_RS05440	NZ_CP043953.1	1,159,945–1,160,214	TTAAttCAAATTGGTCTGGAGCCTA	270	150
				gttaaTaGGTGTCCAAACAGTACATAC		150
ECC	*sufA*	NZ_OW968328.1	1,993,105–1,993,431	tcTGGAACTGCATTCAGAAACC	327	150
				gtCGGGTTATGAWATTTGAATAACTGGT		150
huDNA	RPL37A	NC_000002.12	216,503,028–216,503,322	tcTAGGACCAGGAGTGACAAGT	295	150
				gtAATCCAAACACCCAGCATATAC		150
IC	–	–	–	CGCAATGGTCTTGTCTCATA	347	150
				gtAGGTACAATTAGCTCATCCCTA		150

^*a*^ huDNA, human DNA; IC, internal control; AB, *Acinetobacter baumannii*; KP, *Klebsiella pneumoniae*; ECO, *Escherichia coli*; ECC, *Enterobacter cloacae complex*; SM*, Stenotrophomonas maltophilia*; PA, *Pseudomonas aeruginosa*; SA, *Staphylococcus aureus*; MRSA, methicillin-resistant *Staphylococcus aureus*; SPN, *Streptococcus pneumoniae*; SPY, *Streptococcus pyogenes*; HI, *Haemophilus influenzae*; MC*, Moraxella catarrhalis*; LP, *Legionella pneumophila*.

### Establishment of the MPCE detection system

Multiplex PCR amplification was performed in a final volume of 20 µL containing 8 µL of reaction mixture, 2 µL of enzyme mixture, and 10 µL of nucleic acid extracts. The reaction mixture for 50 reactions contained 250 µL of 4× reaction buffer (deoxyribonucleoside triphosphates (dNTPs ), Mg^2+^, and Tris-HCl buffer) (Haiyi Gene Technology Co., Ningbo), 24.5 µL of each of the 100 µM forward and reverse primers (final concentration shown in Table 1), 51 µL of TE buffer, and 50 µL of nuclease-free water. The enzyme mixture for the 50 reactions contained 80 µL of hot-start DNA polymerase and 20 µL of UDG enzyme (Haiyi Gene Technology Co., Ningbo). The thermal cycling conditions were as follows: 25°C for 150 s, 95°C for 120 s, one cycle; 94°C for 20 s, 59°C for 45 s, 30 cycles; and 60°C for 120 s, one cycle. The fragment sizes of the amplification products are shown in Table 1. One microliter of PCR products and 9 µL of Hi-Di formamide injection solvent containing 2.5% fluorescent internal standard dye (Health Gene, Ningbo) were mixed evenly and added to the fully automatic capillary electrophoresis instrument CE2400 (Health Gene, Ningbo). The PCR amplification products were separated by capillary electrophoresis, and PCR products of different fragment sizes were separated. By detecting the fluorescence signal intensity (i.e., peak height values) of different fragments, the pathogens can be identified.

### Construction of peak maps for standard strains via MPCE and threshold setting via ROC curve plotting

Standard strains included *A. baumannii* BNCC194496, *K. pneumoniae* BNCC102997, *E. coli* BNCC133264, *E. cloacae complex* (*E. cloacae* BNCC336662, *Enterobacter asburiae* BNCC188016, *Enterobacter hormaechei* BNCC358259, *Enterobacter kobei* BNCC358237, and *Enterobacter ludwigii* BNCC120124), *S. maltophilia* BNCC185982, *P. aeruginosa* BNCC340634, *S. aureus* BNCC186335, methicillin-resistant *S. aureus* BNCC337371, *S. pneumoniae* BNCC338425, *S. pyogenes* BNCC337110, *H. influenzae* BNCC259887, *M. catarrhalis* BNCC337550, and *L. pneumophila* BNCC319755 (BNCC, Beijing). The target genes of the 13 standard strains were amplified via the primers listed in Table 1. The amplified products were subjected to capillary electrophoresis to obtain the detection peak maps of each target gene. The highly conserved sequences of standard strains were individually amplified via the primers in Table 2, and the products were recovered via agarose gel electrophoresis and gel cutting, subjected to Sanger sequencing, and verified via BLAST comparison analysis. A total of 101 clinical sputum samples were included in the test, and the peak height value of each target was determined for all the samples. The receiver operating characteristic (ROC) curve was plotted on the basis of the MPCE detection results and Sanger sequencing results, and the Youden index was calculated (Youden index = sensitivity + specificity − 1). The value corresponding to the maximum Youden index was set as the optimal threshold.

**TABLE 2:** Sanger sequencing primer sequences

Forward primer names	Primers sequence (5′–3′)	Reverse primer names	Primer sequence (5′–3′)
MRSA-F	AGGGTTTTCCCAGTCACGATAAATCTTGGGGTGGTTACAACGTTA	MRSA-R	GAGCGGATAACAATTTCACACACACTTTACCTGAGATTTTGGCATT
AB-F	AGGGTTTTCCCAGTCACGGGACACAATGCAAGCGAAAT	AB-R	GAGCGGATAACAATTTCACACGGCTGCAATACTAAAGACACC
ECO-F	AGGGTTTTCCCAGTCACGATATTGAACCGCAGGAAATTGACAG	ECO-R	GAGCGGATAACAATTTCACACACGCATCCATTAATTTGTCATACCTT
SPY-F	AGGGTTTTCCCAGTCACGGTTCTGGACAACACCCGAGT	SPY-R	GAGCGGATAACAATTTCACACGCTTCAATAATAGCCATGCGAACA
SPN-F	AGGGTTTTCCCAGTCACGAAAGCTCACGTTTATGCTATCCCT	SPN-R	GAGCGGATAACAATTTCACACCTAATAGCCAGAAGTTTGCCAAGGT
HI-F	AGGGTTTTCCCAGTCACGACTTGCCTTGAAATGACAAAATTAGGTT	HI-R	GAGCGGATAACAATTTCACACATCTAGGTTTTCTCCGCAAGTGA
KP-F	AGGGTTTTCCCAGTCACGCCTTTGTTGCTTGCCAATGTCT	KP-R	GAGCGGATAACAATTTCACACCGCAGCATAAATAAAATTGCCCAT
SA-F	AGGGTTTTCCCAGTCACGACTTGTACGCATGAAAGTTATAACGA	SA-R	GAGCGGATAACAATTTCACACTGCAATTCTTGTTGTAACTTTGCTGTTC
LP-F	AGGGTTTTCCCAGTCACGTGGACAATCTATCGCCAATGCTAC	LP-R	GAGCGGATAACAATTTCACACACGGATGATATCTCTAATAGCCCCT
MC-F	AGGGTTTTCCCAGTCACGATCACTCGCTCAGGTTCAACTTGG	MC-R	GAGCGGATAACAATTTCACACATGGCGAAACATTGTCAGATAAGGTA
SM-F	AGGGTTTTCCCAGTCACGGCTATAAAATCCGCGAGCATACGTTG	SM-R	GAGCGGATAACAATTTCACACATCTGGTAGTCGCCCTCGTC
PA-F	AGGGTTTTCCCAGTCACGGTAGCGGCGGTCGATCATCA	PA-R	GAGCGGATAACAATTTCACACTGGTTAAATGTAATAGCGAGAACCTG
ECC-F	AGGGTTTTCCCAGTCACGCGCTCCAGGTATTTACATCACTCGT	ECC-R	GAGCGGATAACAATTTCACACATGACCGTTCTGCGTTATGCC

### Sensitivity, specificity, and reproducibility analysis of MPCE

#### Sensitivity

The standard strains were diluted with negative sputum samples at a fivefold gradient, for a total of three gradients. Each gradient was extracted and detected (three batches of reagents, three replicates per batch), with the lowest detectable concentration at 100% (all detected in three replicates) as the estimated detection limit. Negative sputum samples were diluted with a twofold concentration gradient near the estimated detection limit of each target, for a total of three gradients. Each dilution gradient (three batches of reagents, 20 replicates per batch) was extracted and detected. The lowest concentration level with a positive detection rate of ≥95% (at least 19 replicates per batch) was used as the determined limit of detection (LOD) to evaluate the sensitivity of the MPCE detection method.

#### Cross-specificity

Pathogens commonly found in the respiratory tract or those that are prone to cause similar clinical symptoms, as well as nearby pathogens targeted for detection, were detected to evaluate the cross-specificity of the MPCE method. The viruses were diluted to 10^5^ cps/mL, and the bacteria were diluted to 10^6^ cfu/mL. The viruses included respiratory syncytial virus type A, respiratory syncytial virus type B, human parainfluenza virus type 2, human coronavirus 229E, and adenovirus type 1. The bacteria included *Streptococcus dysgalactiae*, *Streptococcus sanguinis*, *Streptococcus mutans*, *Streptococcus salivarius*, *Staphylococcus epidermidis*, *Staphylococcus haemolyticus, Staphylococcus hominis*, *Staphylococcus lugdunensis*, *Staphylococcus schleiferi*, *Staphylococcus saprophyticus*, *Klebsiella oxytoca*, *Klebsiella aerogenes*, *Klebsiella terrigena*, *Pseudomonas alcaligenes*, *Pseudomonas fluorescens*, *Pseudomonas stutzeri*, *Pseudomonas pseudoalcaligenes*, *Acinetobacter junii*, *Acinetobacter calcoaceticus*, *Acinetobacter lwoffii*, *Stenotrophomonas acidaminiphila*, *Alcaligenes faecalis*, *Micrococcus luteus*, *Enterococcus faecalis*, *Serratia marcescens*, and *Proteus vulgaris*.

#### Interfering substances

Common drugs that may exist in the sample or endogenous and exogenous substances that may exist in the samples were used as interfering substances in this experiment. All target bacteria were diluted with negative sputum samples, and each sputum sample contained multiple bacteria to cover all targets. Each interfering substance was added to prepare mixed samples with final bacterial concentrations of two moderately positive (6 LOD) and two weakly positive (1.5 LOD). Samples without interfering substances were used as controls. Interfering substances, including human genomic DNA, whole blood, mucin, azithromycin, cefuroxime, mupirocin, zanamivir, ribavirin, oseltamivir, peramivir, phenylephrine hydrochloride, and oxymetazoline, were evaluated to determine whether they affected the results. The interference of high-concentration pathogens on low-concentration pathogens was evaluated; the high concentration was set to 100 LOD, and the low concentration was set to 1.5 LOD. Negative sputum was prepared into common combinations of complex infections, with lower concentrations of individual pathogens as a control. The impact of competitive interference was evaluated by detecting samples of complex infections and controls.

#### Reproducibility

Two experimenters used testing reagents daily to test five simulated samples at different levels, including two moderately positive samples, two weakly positive samples, and one negative sample. Two complete tests were completed daily for a total of 20 working days to evaluate the reproducibility of the MPCE method.

### The clinical detection ability of MPCE

Qualified sputum samples from patients with pneumonia or pulmonary infection were collected, and appropriate culture media were selected for the sputum culture of 13 bacteria. If bacteria grew on the culture media, a VITEK MS IVD 3.0 automatic rapid microbial mass spectrometry identification system (BioMérieux, France) was used for bacterial identification. The culture method was used as a reference method to evaluate the clinical performance of the MPCE. When the results of the MPCE method and the culture method were inconsistent, the samples were sent for Sanger sequencing verification if the results were still inconsistent after repeated MPCE analysis.

### Result judgment

Fifteen characteristic peaks (13 pathogen characteristic peaks, a human DNA peak, and an internal control peak) appeared in the positive control, with peak heights ≥ threshold. The actual fragment size of each target feature peak should be within a deviation range of ±1.5 nt from the reference fragment size. The human DNA peak and internal control peak must appear in the negative control, with peak heights ≥ threshold.

Positive result determination: If the characteristic peak of the pathogen was ≥threshold, the result was determined to be positive.

Negative result determination: When there was no pathogen characteristic peak or the peak height <threshold: (i) if there were human DNA peak and internal control peak with peak heights ≥ threshold, the result was determined to be negative; (ii) if there was no human DNA peak or the peak height < threshold, it was considered that the sample was taken or stored improperly, and a new sample should be taken for extraction and testing; and (iii) if there was no internal control peak or the peak height < threshold, it was considered that the detection failed and that the sample should be re-extracted and tested.

### Statistical analysis

Count data were expressed as percentages (%), and the results of MPCE and culture methods were analyzed via Kappa values and McNemar’s tests. The consistency of the test results between the two methods was evaluated by the Kappa value. The Kappa value ranges from −1 to 1; the closer the value is to 1, the greater the consistency of the results between the two methods. Differences in test results between the two methods were assessed via McNemar’s test. Statistical analysis was conducted via SPSS 19.0 software, with *P* < 0.05 indicating statistically significant differences.

## RESULTS

### Peak maps of the standard strains and threshold results

**Fig 1:**
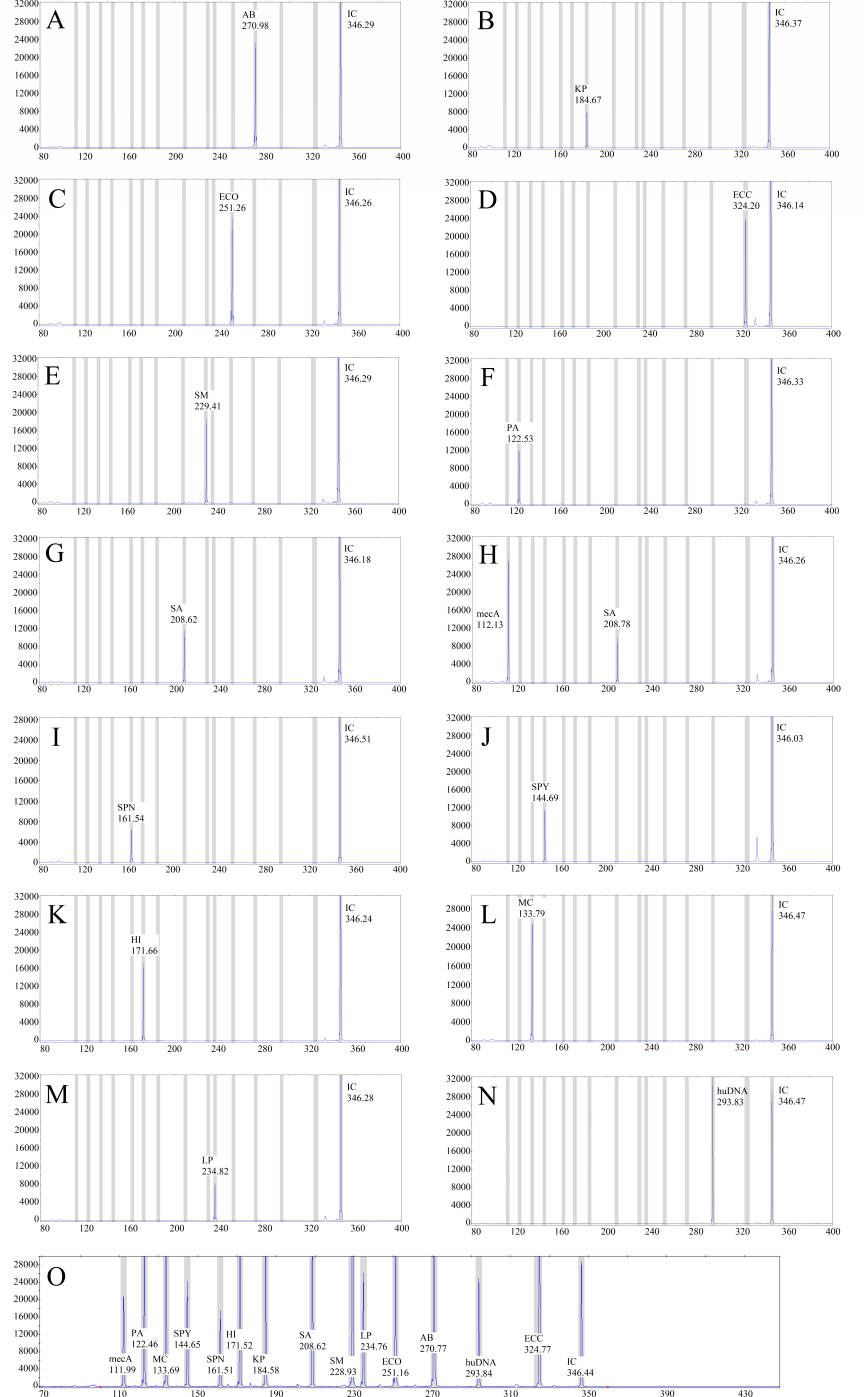
Map of standard strains and positive/negative controls detected via MPCE. The *X*-axis indicates the PCR product size (nt), and the *Y*-axis indicates the fluorescence signal intensity (RFU). (**A**) Result for *Acinetobacter baumannii*. (**B**) Result for *Klebsiella pneumoniae*. (**C**) Result for *Escherichia coli*. (**D**) Result for *Enterobacter cloacae complex*. (**E**) Result for *Stenotrophomonas maltophilia*. (**F**) Result for *Pseudomonas aeruginosa*. (**G**) Result for *Staphylococcus aureus*. (**H**) Result for methicillin-resistant *S. aureus*. (**I**) Result for *Streptococcus pneumoniae*. (**J**) Result for *Streptococcus pyogenes*. (**K**) Result for *Haemophilus influenzae*. (**L**) Result for *Moraxella catarrhalis*. (**M**) Result for *Legionella pneumophila*. (**N**) Result of the negative control. (**O**) Result of the positive control.

The MPCE method was used for standard strain detection, and the peak maps of each target gene were obtained, as shown in Fig. 1. There were 15 characteristic peaks (13 pathogen characteristic peaks, a human DNA peak, and an internal control peak) in the positive control, with peak heights ≥ 1,000 RFU. The human DNA peak and internal control peak appeared in the negative control with peak heights ≥ 1,000 RFU. The size of each target on the peak maps varies within 1.5 nt after repeated testing. The ROC curve (Fig. S1) was plotted on the basis of the MPCE detection results (Table S1) and Sanger sequencing results. According to the ROC curve coordinates, when the threshold was 963.50 RFU, the corresponding Youden index was 0.996, and when the threshold was 1,071.50 RFU, the corresponding Youden index was 0.992 (Table S2). Therefore, the final positive judgment value was set to 1,000 RFU.

### Sensitivity analysis results of MPCE

The sensitivity analysis results of the MPCE method are shown in Table 3. The minimum detection limit range for all tested pathogen targets was 6.0 × 10^3^ cfu/mL~2.0 × 10^6^ cfu/mL.

**TABLE 3:** MPCE detection limits for each pathogen target gene

Targets	Gene	Detection limits (cfu/mL)
AB	F3P16_RS05440	5.0 × 10^5^
KP	KPHS_16050	4.5 × 10^4^
ECO	*oxc*	5.0 × 10^5^
ECC	*sufA*	2.5 × 10^5^
SM	DQN92_RS09665	5.0 × 10^5^
PA	PA1767	1.5 × 10^4^
SA	SAOUHSC_01837	2.0 × 10^6^
MRSA	*mecA*	4.0 × 10^5^
SPN	SPNHU17_RS05160	6.0 × 10^3^
SPY	*speB*	1.0 × 10^4^
HI	*fucA*	4.0 × 10^5^
MC	*lepA*	1.0 × 10^4^
LP	*wipC*	3.0 × 10^4^

### Specificity analysis results of MPCE

When other pathogens, including multiple viruses and bacteria, were detected, all the test results were negative. The results indicated that there was no cross-reactivity between the target pathogens and other pathogens commonly found in the respiratory tract or easily causing similar clinical symptoms or that close source pathogens were detected in the target areas via the MPCE detection method. When the sputum sample contained human genomic DNA within 33 ng/µL, human whole blood with a concentration of less than 30%, mucin within 2.5 mg/mL, azithromycin within 8 ng/mL, cefotaxime within 9 µg/mL, mupirocin within 6 ng/mL, zanamivir within 426 ng/mL, ribavirin within 60 mg/mL, oseltamivir within 7.5 mg/mL, peramivir within 0.2 mg/mL, phenylephrine hydrochloride within 0.3 mg/mL, and oxymetazoline within 25 ng/mL, the result was still positive, and the peak height was not significantly different from that of the control. The results of low-concentration bacteria were still positive after high-concentration interfering strains were added, and the peak height value was not significantly different from that of the control.

### Reproducibility analysis results of MPCE

The results of the reproducibility analysis of the MPCE method revealed that the intra-assay and inter-assay reproducibility for moderately positive samples and weakly positive samples reached 100%, and the intra-assay and inter-assay reproducibility for negative samples reached 100%.

### The clinical detection ability of MPCE

A total of 420 qualified lower respiratory sputum samples were included, with 256 culture-positive samples and 164 culture-negative samples (Table 4). When the culture method was used as the reference, among the 256 culture-positive samples, the results revealed that eight samples for MPCE detection were inconsistent with the culture method, with an overall sensitivity of 96.9%. The sensitivity of the MPCE method for detecting *A. baumannii, E. coli, E. cloacae complex, S. maltophilia, S. aureus, S. pneumoniae, H. influenzae,* and *L. pneumo*phila was 100%. The sensitivity for *K. pneumoniae* was 95.6% (43/45), and two cases were not detected; one case was not detected by Sanger sequencing, and the other case had a peak height of 939 RFU. The sensitivity for *P. aeruginosa* was 95.3% (41/43), with one case not detected by Sanger sequencing and another case with a peak height of 421 RFU. The sensitivity for methicillin-resistant *S. aureus* was 91.7% (11/12), with one case not detected by Sanger sequencing. The sensitivity for *S. pyogenes* was 80% (8/10), with one case not detected by Sanger sequencing and another case with a peak height of 804 RFU. The sensitivity for *M. catarrhalis* was 88.9% (8/9), with one case with a peak height of 841 RFU. For samples with a peak height of less than 1,000 RFU detected by MPCE, the culture results revealed that bacterial colonies only grew in the first zone of the culture medium, with a colony count of less than 10 cfu.

**TABLE 4:** Sensitivity and specificity of MPCE for detecting clinical samples

Targets	Number of culture positive	Number of MPCE positive	Sensitivity	Number of culture negative	Number of MPCE negative	Specificity	Percentage of agreement (%)	Kappa value	McNemar *P-*value
AB	26	26	100%	164	149	90.9%	92.1%	0.731	0.000
KP	45	43	95.6%	164	136	82.9%	85.6%	0.649	0.000
ECO	23	23	100%	164	160	97.6%	97.9%	0.908	0.125
ECC	16	16	100%	164	161	98.2%	98.3%	0.905	0.250
SM	23	23	100%	164	115	70.1%	73.8%	0.366	0.000
PA	43	41	95.3%	164	143	87.2%	88.9%	0.710	0.000
SA	21	21	100%	164	159	97.0%	97.3%	0.878	0.063
MRSA	12	11	91.7%	164	159	97.0%	96.6%	0.768	0.219
SPN	14	14	100%	164	110	67.1%	69.7%	0.243	0.000
SPY	10	8	80%	164	164	100%	98.9%	0.883	0.500
HI	11	11	100%	164	157	95.7%	96.0%	0.738	0.016
MC	9	8	88.9%	164	162	98.8%	98.3%	0.833	1.000
LP	3	3	100%	164	164	100%	100%	1.000	1.000

Using the culture method as the reference, among the 164 culture-negative samples, the MPCE method had a specificity of >90% for detecting *A. baumannii*, *E. coli*, *E. cloacae complex*, *S. aureus*, methicillin-resistant *S. aureus*, *S. pyogenes*, *H. influenzae*, *M. catarrhalis*, and *L. pneumophila*. The specificity for detecting *K. pneumoniae* and *P. aeruginosa* was 80%–90%. The specificity for detecting *S. maltophilia* and *S. pneumoniae* was <80% (Table 4). The inconsistent samples were sent for Sanger sequencing, and the results showed that the MPCE detection results were consistent with the Sanger sequencing results.

The percentage of agreement between the two methods for detecting pathogens ranged from 69.7% to 100%. The agreement rate for *A. baumannii*, *E. coli*, *E. cloacae complex*, *S. aureus*, methicillin-resistant *S. aureus*, *S. pyogenes*, *H. influenzae*, *M. catarrhalis*, and *L. pneumophila* was >90%. The Kappa values for *A. baumannii*, *K. pneumoniae*, *E. coli*, *E. cloacae complex*, *P. aeruginosa*, *S. aureus*, methicillin-resistant *S. aureus*, *S. pyogenes*, *H. influenzae*, *M. catarrhalis*, and *L. pneumophila* were >0.61, which was highly consistent with the culture method. There was no statistically significant difference between the MPCE method and the culture method in the detection of *E. coli*, *E. cloacae complex*, *S. aureus,* methicillin-resistant *S. aureus*, *S. pyogenes*, *M. catarrhalis*, or *L. pneumophila* (*P* > 0.05).

## DISCUSSION

Pneumonia is an important global health issue associated with significant morbidity, mortality, and increased treatment costs (1). Hospital-acquired pneumonia is the second most common nosocomial infection, with the highest incidence rate among immunocompromised patients, elderly patients, and surgical patients, and is the main cause of death from nosocomial infection in critically ill patients (9, 10). Nosocomial pneumonia is often caused by multidrug-resistant microorganisms (11), which increases the difficulty of treating pneumonia, prolongs hospital stays, increases medical costs, and increases incidence rates and mortality. Therefore, the diagnosis and treatment of pneumonia require the rapid and accurate detection of infectious pathogens. The traditional methods for detecting pathogens include the use of smears and cultures of sputum or bronchoalveolar lavage fluid, as well as the detection of serum biomarkers. Currently, sputum culture combined with sputum smear is widely used in clinical laboratory testing as a basis for the clinical diagnosis of lower respiratory tract bacterial infections. However, when the culture method or medium selected by the microbiology laboratory is not conducive to the growth of pathogens, the influence of antibacterial drugs, the extension of sample submission time, and improper storage conditions may lead to false negative results, especially those affecting subsequent treatment. Obviously, the culture method can only partially meet clinical needs. Therefore, rapid and accurate identification of the pathogens involved in bacterial pneumonia will contribute to early clinical diagnosis and sufficient empirical treatment.

In this study, 13 bacteria, which are important pathogens that cause bacterial pneumonia, were selected as detection targets for pneumonia. *S. pneumoniae*, *S. aureus*, *H. influenzae,* and *A. baumannii* can cause necrotizing pneumonia (12–14). Both *S. pneumoniae* and *L. pneumophila* can cause pulmonary consolidation (15, 16). Among the pathogens causing ventilator-associated pneumonia, *P. aeruginosa* was identified as the main pathogen in 29.2%, followed by methicillin-resistant *S. aureus* (12.0%) and *K. pneumoniae* (9.5%), and the detection rate of multidrug-resistant pathogens reached 57.8% (17, 18). *S. maltophilia* can cause hemorrhagic pneumonia in patients with hematological malignancies (19). The isolation rate of *S. maltophilia* in pneumonia patients in ICUs is 16.3%, and the mortality rate is relatively high (20, 21). *P. aeruginosa*, *A. baumannii*, *K. pneumoniae*, *E. coli*, *Enterobacter*, *S. maltophilia*, and *S. aureus* are common pathogens that cause lower respiratory tract infections in Asian countries (1, 22). Among them, *A. baumannii*, *K. pneumoniae*, *E. coli*, *P. aeruginosa*, and *S. aureus isolates* are mostly typical multidrug-resistant bacteria (23), which can cause the spread of nosocomial infections. *S. pneumoniae*, *S. pyogenes*, *H. influenzae*, *M. catarrhalis*, and *L. pneumophila* are pathogens that are difficult to culture or prone to missed detection.

The MPCE detection method has high sensitivity and specificity and is based on multiplex PCR amplification and the isolation of amplification products of different lengths by capillary electrophoresis, which is a fast and reliable nucleic acid detection technology. We report a study using the MPCE method in detecting multiple respiratory pathogens, which is a rapid molecular test for identifying respiratory pathogens. We selected 13 bacteria as detection targets, and the MPCE detection system we studied included 15 different pairs of primers (13 target pathogens, human DNA, and internal control). Each pair of primers amplified a pathogen fragment, and different amplification products had different lengths. When a capillary electrophoresis analyzer is used to analyze amplification products, smaller fragments move faster, and larger fragments move slower. By comparing the migration time with the size standard, various lengths of PCR product fragments were determined to achieve the simultaneous detection of 13 bacterial species. This method significantly reduces the detection time of clinical pathogenic bacteria and improves detection efficiency. The MPCE reaction system in this study contained the UDG enzyme, which effectively prevented the contamination of the amplification products. Human DNA in a sample can be detected to monitor sample quality. If inhibitors are present in clinical samples, the quality of PCR amplification may decrease. Therefore, internal control was included in each amplification reaction to amplify the target DNA in the clinical sample to monitor the entire detection process of nucleic acid extraction, PCR amplification, and capillary electrophoresis. The MPCE reaction system has an anticontamination system and dual-quality control to ensure more accurate detection results.

Previous studies have shown that the capillary electrophoresis separation method has been clinically applied for the detection of sexually transmitted diseases, human papillomaviruses, influenza viruses, and atypical bacteria such as *M. pneumoniae* (24–27). The sensitivity and specificity of capillary electrophoresis for *Mycoplasma genitalium*, *Mycoplasma hominis*, *Mycoplasma urealyticum*, *Ureaplasma parvum*, *Chlamydia trachomatis*, *Neisseria gonorrhoeae*, and *Trichomonas vaginalis* are 98%–100% and 97%–100%, respectively. Six clinically important species of *Candida* can be quickly identified, with 100% specificity in identifying *Candida albicans*, *Candida krusei*, *Candida parapsilosis*, *Candida glabrata*, *Candida tropicalis*, and *Candida dubliniensis* (28). This method has also been used to identify eight important foodborne microorganisms (*E. coli*, *Clostridium perfringens*, *Campylobacter jejuni*, *Salmonella enterica*, *Listeria monocytogenes*, *Vibrio parahaemolyticus*, *S. aureus*, *and Bacillus cereus*) (29) and detect nine pathogenic viruses in pigs (30). This study is the first to use MPCE for the detection of 13 pathogens in bacterial pneumonia patients. As the proportion of mixed infections caused by multiple pathogens increases in critically ill patients, organ transplant patients, and immunocompromised patients (31, 32), incurable chronic and persistent pulmonary infections may induce more severe hyperinflammatory syndrome, hyperinflammatory shock, and higher mortality. Delaying effective treatment increases in-hospital mortality in pneumonia patients, making the selection of empirical drugs a key dilemma (33). To increase the possibility of sufficient coverage, it is increasingly necessary to detect multiple pathogens. However, current culture methods or single-detection PCRs are cumbersome and costly, making them time-consuming and expensive. The MPCE method, which is based on multiplex PCR, can save considerable time and cost. This assay can simultaneously identify 13 pathogens closely related to bacterial pneumonia in 2.5 h, which is significantly faster than the culture method and reduces the number of required samples, which is particularly important when samples are limited. The respiratory pathogen nucleic acid detection kit (multiplex fluorescence PCR method) currently used in clinical practice in China can simultaneously detect six pathogens, whereas the respiratory pathogen nucleic acid detection kit (isothermal amplification chip method) can detect seven pathogens. This MPCE method can simultaneously detect 13 pathogens. Moreover, as this method does not require probes, the cost of reagents required for each sample is only ¥25–¥30 ($3.5–$4.2), which is superior to the currently available multiplex fluorescence PCR assay kits and isothermal amplification chip method kits on the domestic market. MPCE will become an important technical means for the identification and screening of pathogenic bacteria associated with infectious diseases.

In the MPCE study, peak maps were drawn via standard strains, and the positive judgment threshold was set to ≥1,000 RFU on the basis of the ROC curve. The performance evaluation of MPCE using standard strains revealed a minimum detection limit range of 6.0 × 10^3^ cfu/mL~2.0 × 10^6^ cfu/mL for detecting 13 bacteria. There was no cross-reactivity with other pathogens commonly found in the respiratory tract or easily causing similar clinical symptoms or close source pathogens with strong specificity. A total of 420 clinical samples were used for evaluation, with sputum culture results used as a reference. Among the 256 culture-positive samples, eight samples tested negative for MPCE. Among them, one case of *K. pneumoniae*, one case of *P. aeruginosa,* one case of methicillin-resistant *S. aureus*, and one case of *S. pyogenes* were subjected to Sanger sequencing, and the sequencing results were consistent with those of MPCE, considering that the nucleic acids of some pathogens may be degraded before detection due to temperature changes during storage. One case of *K. pneumoniae*, one case of *P. aeruginosa*, one case of *S. pyogenes*, and one case of *M. catarrhalis* were judged negative because the peak height was <1,000 RFU, and the culture results of the four samples revealed a small number of bacteria. This false negative was likely caused by the small number of bacteria in the samples, which was lower than the detection limit of MPCE, and the primer concentration should be adjusted for these pathogens in future studies. For 164 culture-negative samples, the MPCE method achieved 100% specificity for *S. pyogenes* and *L. pneumophila*, and the specificities for *E. coli*, *E. cloacae complex*, *S. aureus*, methicillin-resistant *S. aureus*, *H. influenzae*, and *M. catarrhalis* were all >95%. Inconsistent samples were subjected to Sanger sequencing, and the sequencing results were consistent with the MPCE results. MPCE has an important value for culture-negative samples and can significantly improve the detection rate. For pathogens that are inhibited by antibiotics but not completely killed, the culture results may be false negatives, leading to missed detection. Nevertheless, MPCE can still detect these pathogens.

There are several limitations in this study. Although the cost of MPCE detection is significantly lower than that of Sanger sequencing, it has the same problem as Sanger sequencing. While detecting infections, it can also detect residual or dead bacterial undecomposed DNA in samples, leading to false positives. MPCE, Sanger sequencing, and culture methods have the same problem of needing help to distinguish between colonization and infection. Owing to oropharyngeal colonization bacteria such as *S. pneumoniae* (25), the specificity of certain bacteria in clinical evaluation is relatively low compared with that of the culture method. Therefore, more qualified respiratory samples, such as bronchoalveolar lavage fluid samples, are needed for clinical evaluation.

This study successfully established an MPCE detection system that can simultaneously detect 13 pathogens through a single detection method, significantly improving clinical efficiency. It is also suitable for patients at risk of infection with multiple pathogens, providing a fast, accurate, and high-throughput detection method for the diagnosis of bacterial pneumonia.

## SUPPLEMENTAL MATERIAL

Figure S1; Tables S1 and S2 spectrum.01202-24-s0001.doc ROC curve and Youden index.

ASM does not own the copyrights to Supplemental Material that may be linked to, or accessed through, an article. The authors have granted ASM a non-exclusive, world-wide license to publish the Supplemental Material files. Please contact the corresponding author directly for reuse.
