# Supplementary material for: Establishment and evaluation of a rapid method for the detection of bacterial pneumonia in hospitalized patients via multiplex PCR–capillary electrophoresis (MPCE)
Source: Microbiol Spectr. 2024 Sep 18;12(11):e01202-24. doi: 10.1128/spectrum.01202-24 (PMC11537078; doi:10.1128/spectrum.01202-24)
Supplement: Figure S1; Tables S1 and S2 — ROC curve and Youden index. [file spectrum.01202-24-s0001.doc]

**
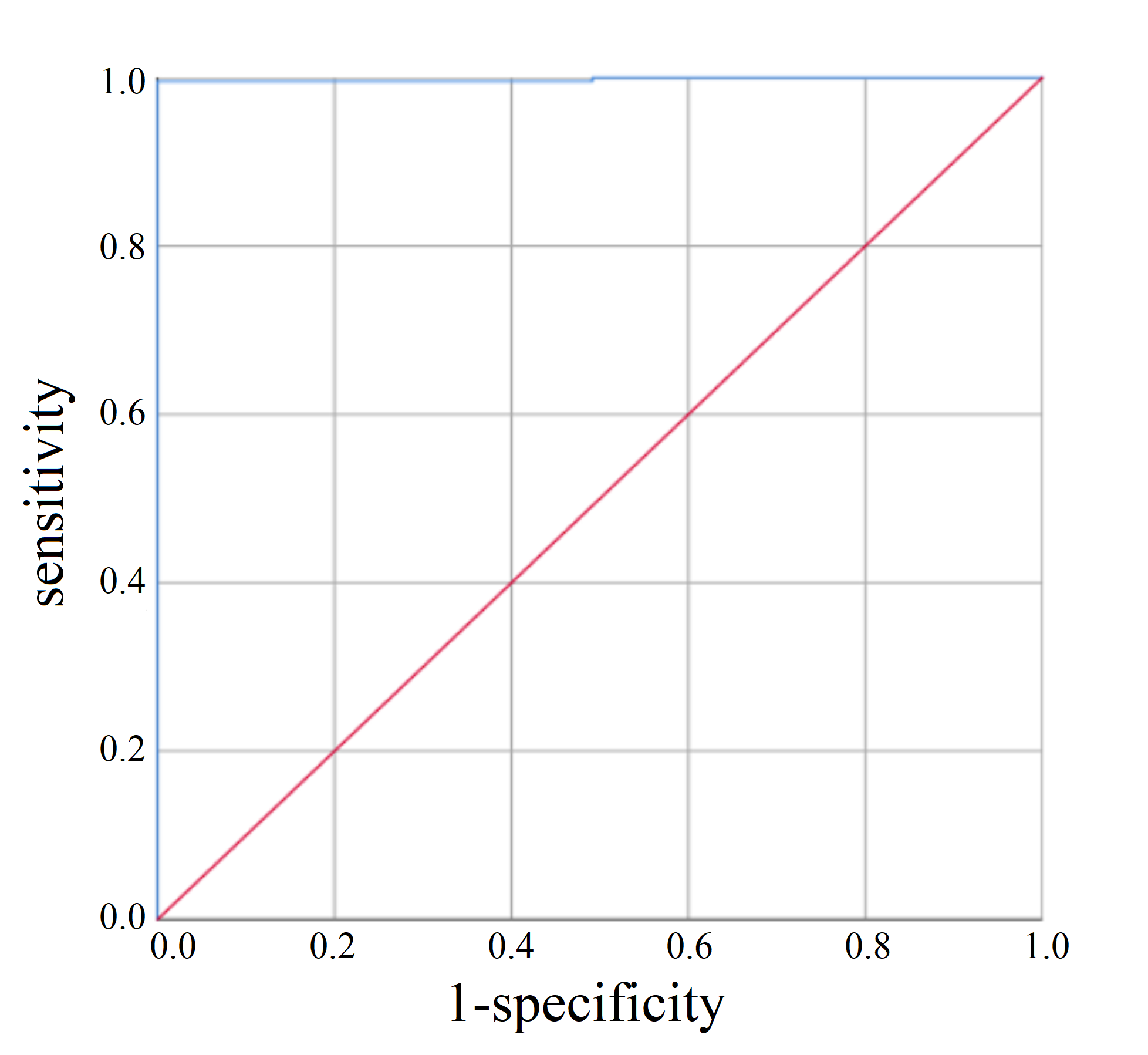
**

**Figure S1 ROC curve chart**

**Table S1 Peak height value of 101 clinical sputum samples**

| Number | Peak height value (RFU) | | | | | | | | | | | | |
| --- | --- | --- | --- | --- | --- | --- | --- | --- | --- | --- | --- | --- | --- |
|  | SM | ECC | PA | SPY | mecA | SPN | KP | LP | AB | ECO | HI | SA | MC |
| 1 | 32473 | 32539 | - | - | - | - | 14548 | - | 32463 | - | - | - | - |
| 2 | 32270 | 32182 | - | - | 4433 | 32366 | - | - | 32168 | - | - | - | - |
| 3 | 13747 | 32377 | - | - | - | 15658 | - | - | 21954 | - | - | - | 6620 |
| 4 | - | - | - | - | - | 31594 | - | - | - | - | - | 549 | - |
| 5 | - | - | - | - | 389 | 32245 | - | - | - | - | - | - | - |
| 6 | - | - | 32372 | - | 11667 | 20253 | - | - | - | - | - | 11729 | - |
| 7 | - | - | - | - | - | - | - | - | - | - | - | - | - |
| 8 | - | - | - | - | 31916 | 32162 | - | - | - | - | - | 32317 | - |
| 9 | 391 | - | - | - | 12583 | - | - | - | - | - | - | 13337 | - |
| 10 | - | - | - | - | - | 32357 | - | - | - | - | - | - | 32330 |
| 11 | - | - | - | - | 5831 | - | - | - | 32440 | - | 4339 | - | - |
| 12 | - | 32422 | 32368 | - | 9733 | - | - | - | - | - | - | - | - |
| 13 | - | - | - | - | 32099 | - | - | - | - | - | - | - | - |
| 14 | 7392 | - | 32361 | - | 10151 | - | - | - | - | - | - | - | - |
| 15 | 554 | - | - | - | 541 | 11353 | - | - | - | - | - | - | - |
| 16 | 17680 | - | - | - | 20360 | 14716 | 31535 | - | - | - | - | - | - |
| 17 | - | - | 32428 | - | 6466 | - | 21404 | - | - | - | - | - | - |
| 18 | - | - | - | - | - | 25080 | - | - | - | - | 1672 | - | - |
| 19 | - | - | - | - | - | 29193 | - | - | - | - | 1925 | - | - |
| 20 | - | - | 31700 | - | 32284 | 32242 | 31512 | - | 391 | - | 772 | - | - |
| 21 | - | 32433 | - | - | 10342 | - | 32397 | - | 32374 | - | 7012 | 8514 | - |
| 22 | - | - | - | - | 595 | - | - | - | 767 | - | - | - | - |
| 23 | - | - | - | - | 446 | - | - | - | 526 | - | - | - | - |
| 24 | 32148 | - | 333 | - | 1264 | 32422 | - | - | 32448 | - | - | - | - |
| 25 | 32527 | - | 32340 | - | 8278 | 31876 | 8939 | - | - | - | - | - | - |
| 26 | - | - | - | - | - | 32318 | - | - | - | - | 2259 | - | - |
| 27 | - | - | - | - | - | 32402 | - | - | - | - | 3225 | - | - |
| 28 | - | - | 31962 | - | 32340 | 32254 | 25991 | - | - | - | 613 | - | - |
| 29 | - | 32435 | - | - | 3525 | - | 31634 | - | 32395 | - | 4738 | 3168 | - |
| 30 | - | - | - | - | - | 257 | - | - | - | - | - | - | - |
| 31 | - | - | - | - | - | 229 | - | - | - | - | - | - | - |
| 32 | 4701 | - | - | - | - | 10573 | - | - | 32450 | - | - | - | - |
| 33 | 21262 | - | 32417 | - | 17830 | 14789 | 32453 | - | - | - | - | - | - |
| 34 | - | - | - | - | 32462 | 32552 | - | - | - | - | - | - | - |
| 35 | - | - | - | - | - | - | - | - | - | - | - | - | - |
| 36 | 365 | 784 | - | - | 21759 | 31923 | - | - | - | - | - | 12091 | - |
| 37 | 31558 | - | 31409 | - | 31253 | 31753 | 32014 | - | 437 | - | - | 31919 | - |
| 38 | - | - | - | - | 18368 | 18645 | - | - | 32483 | - | 12882 | - | 495 |
| 39 | - | - | - | - | - | 31703 | - | - | - | - | - | 524 | - |
| 40 | - | - | - | - | - | - | - | - | - | - | - | - | - |
| 41 | - | - | - | 8692 | - | - | - | - | - | - | - | - | - |
| 42 | - | - | - | 30573 | - | - | - | - | - | - | - | - | - |
| 43 | - | - | - | 32385 | - | - | - | - | - | - | - | - | - |
| 44 | 326 | - | 179 | - | 379 | 567 | - | 1931 | - | - | - | 163 | - |
| 45 | 333 | - | - | - | 402 | 424 | - | 32428 | - | - | - | - | - |
| 46 | - | - | - | - | - | - | - | 32439 | - | - | - | - | - |
| 47 | - | - | - | - | - | 11328 | - | - | - | - | - | - | - |
| 48 | - | - | - | - | 32356 | - | 32428 | - | - | - | - | - | - |
| 49 | - | - | - | - | 32364 | 32449 | - | - | - | - | - | - | - |
| 50 | 28694 | - | - | - | - | 22715 | - | - | - | - | 14317 | - | 733 |
| 51 | - | - | - | - | 32311 | 32394 | - | - | - | - | - | - | - |
| 52 | - | - | - | - | - | 32135 | - | - | - | - | - | - | 32148 |
| 53 | 26018 | - | - | - | - | - | - | - | - | - | 14661 | - | 377 |
| 54 | - | - | - | - | - | 150 | - | - | - | - | - | - | - |
| 55 | - | - | - | - | - | 28033 | - | - | - | - | - | - | - |
| 56 | - | - | - | - | - | 16784 | - | - | - | - | - | - | - |
| 57 | - | - | - | - | 441 | 32224 | - | - | - | - | - | 370 | - |
| 58 | - | - | - | - | - | 31517 | - | - | - | - | - | - | - |
| 59 | - | - | - | - | - | 6711 | - | - | 853 | - | - | 7340 | - |
| 60 | - | - | 32427 | - | 4772 | 10161 | - | - | - | - | - | - | - |
| 61 | - | - | - | - | 230 | 235 | - | - | - | - | - | - | - |
| 62 | - | - | - | - | - | - | 32344 | - | - | - | - | - | - |
| 63 | - | - | 32450 | - | - | - | - | - | - | - | - | - | - |
| 64 | - | - | - | - | 530 | 32150 | 327 | - | - | - | - | 418 | - |
| 65 | - | - | 3056 | - | - | 32228 | 734 | - | - | - | - | - | - |
| 66 | - | - | - | - | - | 16081 | - | - | - | - | - | - | - |
| 67 | - | 32405 | - | - | 9249 | - | - | - | - | - | - | - | - |
| 68 | 32344 | 32250 | 422 | - | 31234 | 16545 | 31810 | - | 32299 | - | - | - | - |
| 69 | - | 32437 | - | - | - | 9843 | - | - | - | - | - | - | - |
| 70 | - | 32462 | - | - | - | 8535 | - | - | - | - | - | - | - |
| 71 | 32202 | - | - | - | 32141 | - | 31932 | - | - | - | - | - | - |
| 72 | 19848 | - | - | - | 13026 | 14117 | 32634 | - | 18326 | - | - | - | - |
| 73 | 32103 | - | 31779 | - | 349 | - | - | - | - | - | - | - | - |
| 74 | 323 | - | 3141 | - | - | 32268 | 705 | - | - | - | - | - | - |
| 75 | 13107 | - | 32348 | - | - | 13735 | - | - | - | - | - | - | - |
| 76 | - | - | 32418 | - | - | - | 32411 | - | - | - | - | - | - |
| 77 | 742 | - | 1819 | - | 30894 | 31577 | 32377 | - | - | - | 31954 | - | - |
| 78 | 1542 | 2126 | 1149 | - | 31199 | 6607 | 31911 | - | - | - | 32309 | - | 13988 |
| 79 | - | - | 32400 | - | - | - | - | - | - | - | - | - | - |
| 80 | 17339 | - | 32409 | - | - | 15069 | - | - | - | - | - | - | - |
| 81 | - | - | - | - | 19531 | 22522 | 32348 | - | - | 2101 | 9509 | - | - |
| 82 | 32232 | - | 340 | - | 2204 | 32111 | 19406 | - | - | - | - | - | - |
| 83 | - | - | - | - | 12052 | - | 32337 | - | - | - | - | 9494 | - |
| 84 | - | - | 32375 | - | 9911 | 9911 | 32359 | - | - | - | 7739 | 8618 | - |
| 85 | 32364 | - | - | - | 1578 | 23574 | 13669 | - | - | - | - | - | - |
| 86 | - | - | 32308 | - | 494 | 994 | - | - | - | - | 357 | - | - |
| 87 | - | - | - | - | 2595 | 32394 | 438 | - | - | - | 4890 | - | - |
| 88 | - | - | - | - | 8933 | 10570 | 32430 | - | 21927 | - | - | - | - |
| 89 | - | - | - | - | 484 | 32199 | - | - | - | - | - | - | - |
| 90 | - | - | 32008 | - | - | 1541 | - | - | - | - | 527 | - | - |
| 91 | 933 | - | - | - | - | - | - | - | - | - | - | - | - |
| 92 | 395 | - | - | - | - | - | - | - | - | - | - | - | - |
| 93 | 739 | 385 | - | - | 630 | 14675 | - | - | - | - | - | - | - |
| 94 | - | - | - | - | 12428 | - | 32207 | - | - | - | - | - | - |
| 95 | - | - | - | - | - | - | - | - | - | - | 8317 | - | 351 |
| 96 | 256 | - | - | - | - | - | - | - | - | - | - | - | - |
| 97 | 926 | - | - | - | - | - | - | - | - | - | - | - | - |
| 98 | 31212 | - | 31901 | - | - | - | - | - | 32189 | - | 1883 | - | - |
| 99 | 11525 | - | 28878 | - | 5456 | - | 31696 | - | 32635 | - | - | - | - |
| 100 | 333 | - | - | - | - | - | - | - | - | - | - | - | - |
| 101 | 21902 | - | - | - | 14944 | - | - | - | - | - | - | - | - |

AB（Acinetobacter baumannii）,KP(Klebsiella pneumoniae),ECO(Escherichia coli),ECC( Enterobacter cloacae complex), SM(Stenotrophomonas maltophilia),PA(Pseudomonas aeruginosa),SA(Staphylococcus aureus)，SPN(Streptococcus pneumoniae),SPY(Streptococcus pyogenes),HI(Haemophilus influenzae),MC(Moraxella catarrhalis),LP(Legionella pneumophila ) .

**Table S2 Test result variable of ROC curve**

| Coordinates of the ROC curve | | | |
| --- | --- | --- | --- |
| peak value (RFU)  ( if ≥ this value, it is positive) | sensitivity | 1-specificity | Youden index |
| 149.00 | 1.000 | 1.000 | 0.000 |
| 156.50 | 1.000 | 0.983 | 0.017 |
| 171.00 | 1.000 | 0.966 | 0.034 |
| 204.00 | 1.000 | 0.949 | 0.051 |
| 229.50 | 1.000 | 0.932 | 0.068 |
| 232.50 | 1.000 | 0.915 | 0.085 |
| 245.50 | 1.000 | 0.898 | 0.102 |
| 256.50 | 1.000 | 0.881 | 0.119 |
| 290.00 | 1.000 | 0.864 | 0.136 |
| 324.50 | 1.000 | 0.847 | 0.153 |
| 326.50 | 1.000 | 0.831 | 0.169 |
| 330.00 | 1.000 | 0.814 | 0.186 |
| 336.50 | 1.000 | 0.763 | 0.237 |
| 344.50 | 1.000 | 0.746 | 0.254 |
| 350.00 | 1.000 | 0.729 | 0.271 |
| 354.00 | 1.000 | 0.712 | 0.288 |
| 361.00 | 1.000 | 0.695 | 0.305 |
| 367.50 | 1.000 | 0.678 | 0.322 |
| 373.50 | 1.000 | 0.661 | 0.339 |
| 378.00 | 1.000 | 0.644 | 0.356 |
| 382.00 | 1.000 | 0.627 | 0.373 |
| 387.00 | 1.000 | 0.610 | 0.390 |
| 390.00 | 1.000 | 0.593 | 0.407 |
| 393.00 | 1.000 | 0.559 | 0.441 |
| 398.50 | 1.000 | 0.542 | 0.458 |
| 410.00 | 1.000 | 0.525 | 0.475 |
| 420.00 | 1.000 | 0.508 | 0.492 |
| 423.00 | 1.000 | 0.492 | 0.508 |
| 430.50 | 0.996 | 0.492 | 0.504 |
| 437.50 | 0.996 | 0.475 | 0.521 |
| 439.50 | 0.996 | 0.458 | 0.538 |
| 443.50 | 0.996 | 0.441 | 0.555 |
| 465.00 | 0.996 | 0.424 | 0.572 |
| 489.00 | 0.996 | 0.407 | 0.589 |
| 494.50 | 0.996 | 0.390 | 0.606 |
| 509.50 | 0.996 | 0.373 | 0.623 |
| 525.00 | 0.996 | 0.356 | 0.640 |
| 526.50 | 0.996 | 0.339 | 0.657 |
| 528.50 | 0.996 | 0.322 | 0.674 |
| 535.50 | 0.996 | 0.305 | 0.691 |
| 545.00 | 0.996 | 0.288 | 0.708 |
| 551.50 | 0.996 | 0.271 | 0.725 |
| 560.50 | 0.996 | 0.254 | 0.742 |
| 581.00 | 0.996 | 0.237 | 0.758 |
| 604.00 | 0.996 | 0.220 | 0.775 |
| 621.50 | 0.996 | 0.203 | 0.792 |
| 667.50 | 0.996 | 0.186 | 0.809 |
| 719.00 | 0.996 | 0.169 | 0.826 |
| 733.50 | 0.996 | 0.153 | 0.843 |
| 736.50 | 0.996 | 0.136 | 0.860 |
| 740.50 | 0.996 | 0.119 | 0.877 |
| 754.50 | 0.996 | 0.102 | 0.894 |
| 769.50 | 0.996 | 0.085 | 0.911 |
| 778.00 | 0.996 | 0.068 | 0.928 |
| 818.50 | 0.996 | 0.051 | 0.945 |
| 889.50 | 0.996 | 0.034 | 0.962 |
| 929.50 | 0.996 | 0.017 | 0.979 |
| 963.50 | 0.996 | 0.000 | 0.996 |
| 1071.50 | 0.992 | 0.000 | 0.992 |
| 1206.50 | 0.987 | 0.000 | 0.987 |
| 1402.50 | 0.983 | 0.000 | 0.983 |
| 1541.50 | 0.979 | 0.000 | 0.979 |
| 1560.00 | 0.975 | 0.000 | 0.975 |
| 1625.00 | 0.970 | 0.000 | 0.970 |
| 1745.50 | 0.966 | 0.000 | 0.966 |
| 1851.00 | 0.962 | 0.000 | 0.962 |
| 1904.00 | 0.958 | 0.000 | 0.958 |
| 1928.00 | 0.954 | 0.000 | 0.954 |
| 2016.00 | 0.949 | 0.000 | 0.949 |
| 2113.50 | 0.945 | 0.000 | 0.945 |
| 2165.00 | 0.941 | 0.000 | 0.941 |
| 2231.50 | 0.937 | 0.000 | 0.937 |
| 2427.00 | 0.932 | 0.000 | 0.932 |
| 2825.50 | 0.928 | 0.000 | 0.928 |
| 3098.50 | 0.924 | 0.000 | 0.924 |
| 3154.50 | 0.920 | 0.000 | 0.920 |
| 3196.50 | 0.916 | 0.000 | 0.916 |
| 3375.00 | 0.911 | 0.000 | 0.911 |
| 3932.00 | 0.907 | 0.000 | 0.907 |
| 4386.00 | 0.903 | 0.000 | 0.903 |
| 4567.00 | 0.899 | 0.000 | 0.899 |
| 4719.50 | 0.895 | 0.000 | 0.895 |
| 4755.00 | 0.890 | 0.000 | 0.890 |
| 4831.00 | 0.886 | 0.000 | 0.886 |
| 5173.00 | 0.882 | 0.000 | 0.882 |
| 5643.50 | 0.878 | 0.000 | 0.878 |
| 6148.50 | 0.873 | 0.000 | 0.873 |
| 6536.50 | 0.869 | 0.000 | 0.869 |
| 6613.50 | 0.865 | 0.000 | 0.865 |
| 6665.50 | 0.861 | 0.000 | 0.861 |
| 6861.50 | 0.857 | 0.000 | 0.857 |
| 7176.00 | 0.852 | 0.000 | 0.852 |
| 7366.00 | 0.848 | 0.000 | 0.848 |
| 7565.50 | 0.844 | 0.000 | 0.844 |
| 8008.50 | 0.840 | 0.000 | 0.840 |
| 8297.50 | 0.835 | 0.000 | 0.835 |
| 8415.50 | 0.831 | 0.000 | 0.831 |
| 8524.50 | 0.827 | 0.000 | 0.827 |
| 8576.50 | 0.823 | 0.000 | 0.823 |
| 8655.00 | 0.819 | 0.000 | 0.819 |
| 8812.50 | 0.814 | 0.000 | 0.814 |
| 8936.00 | 0.810 | 0.000 | 0.810 |
| 9094.00 | 0.806 | 0.000 | 0.806 |
| 9371.50 | 0.802 | 0.000 | 0.802 |
| 9501.50 | 0.797 | 0.000 | 0.797 |
| 9621.00 | 0.793 | 0.000 | 0.793 |
| 9788.00 | 0.789 | 0.000 | 0.789 |
| 9877.00 | 0.785 | 0.000 | 0.785 |
| 10031.00 | 0.776 | 0.000 | 0.776 |
| 10156.00 | 0.772 | 0.000 | 0.772 |
| 10251.50 | 0.768 | 0.000 | 0.768 |
| 10456.00 | 0.764 | 0.000 | 0.764 |
| 10571.50 | 0.759 | 0.000 | 0.759 |
| 10950.50 | 0.755 | 0.000 | 0.755 |
| 11340.50 | 0.751 | 0.000 | 0.751 |
| 11439.00 | 0.747 | 0.000 | 0.747 |
| 11596.00 | 0.743 | 0.000 | 0.743 |
| 11698.00 | 0.738 | 0.000 | 0.738 |
| 11890.50 | 0.734 | 0.000 | 0.734 |
| 12071.50 | 0.730 | 0.000 | 0.730 |
| 12259.50 | 0.726 | 0.000 | 0.726 |
| 12505.50 | 0.722 | 0.000 | 0.722 |
| 12732.50 | 0.717 | 0.000 | 0.717 |
| 12954.00 | 0.713 | 0.000 | 0.713 |
| 13066.50 | 0.709 | 0.000 | 0.709 |
| 13222.00 | 0.705 | 0.000 | 0.705 |
| 13503.00 | 0.700 | 0.000 | 0.700 |
| 13702.00 | 0.696 | 0.000 | 0.696 |
| 13741.00 | 0.692 | 0.000 | 0.692 |
| 13867.50 | 0.688 | 0.000 | 0.688 |
| 14052.50 | 0.684 | 0.000 | 0.684 |
| 14217.00 | 0.679 | 0.000 | 0.679 |
| 14432.50 | 0.675 | 0.000 | 0.675 |
| 14604.50 | 0.671 | 0.000 | 0.671 |
| 14668.00 | 0.667 | 0.000 | 0.667 |
| 14695.50 | 0.662 | 0.000 | 0.662 |
| 14752.50 | 0.658 | 0.000 | 0.658 |
| 14866.50 | 0.654 | 0.000 | 0.654 |
| 15006.50 | 0.650 | 0.000 | 0.650 |
| 15363.50 | 0.646 | 0.000 | 0.646 |
| 15869.50 | 0.641 | 0.000 | 0.641 |
| 16313.00 | 0.637 | 0.000 | 0.637 |
| 16664.50 | 0.633 | 0.000 | 0.633 |
| 17061.50 | 0.629 | 0.000 | 0.629 |
| 17509.50 | 0.624 | 0.000 | 0.624 |
| 17755.00 | 0.620 | 0.000 | 0.620 |
| 18078.00 | 0.616 | 0.000 | 0.616 |
| 18347.00 | 0.612 | 0.000 | 0.612 |
| 18506.50 | 0.608 | 0.000 | 0.608 |
| 19025.50 | 0.603 | 0.000 | 0.603 |
| 19468.50 | 0.599 | 0.000 | 0.599 |
| 19689.50 | 0.595 | 0.000 | 0.595 |
| 20050.50 | 0.591 | 0.000 | 0.591 |
| 20306.50 | 0.586 | 0.000 | 0.586 |
| 20811.00 | 0.582 | 0.000 | 0.582 |
| 21333.00 | 0.578 | 0.000 | 0.578 |
| 21581.50 | 0.574 | 0.000 | 0.574 |
| 21830.50 | 0.570 | 0.000 | 0.570 |
| 21914.50 | 0.565 | 0.000 | 0.565 |
| 21940.50 | 0.561 | 0.000 | 0.561 |
| 22238.00 | 0.557 | 0.000 | 0.557 |
| 22618.50 | 0.553 | 0.000 | 0.553 |
| 23144.50 | 0.549 | 0.000 | 0.549 |
| 24327.00 | 0.544 | 0.000 | 0.544 |
| 25535.50 | 0.540 | 0.000 | 0.540 |
| 26004.50 | 0.536 | 0.000 | 0.536 |
| 27025.50 | 0.532 | 0.000 | 0.532 |
| 28363.50 | 0.527 | 0.000 | 0.527 |
| 28786.00 | 0.523 | 0.000 | 0.523 |
| 29035.50 | 0.519 | 0.000 | 0.519 |
| 29883.00 | 0.515 | 0.000 | 0.515 |
| 30733.50 | 0.511 | 0.000 | 0.511 |
| 31046.50 | 0.506 | 0.000 | 0.506 |
| 31205.50 | 0.502 | 0.000 | 0.502 |
| 31223.00 | 0.498 | 0.000 | 0.498 |
| 31243.50 | 0.494 | 0.000 | 0.494 |
| 31331.00 | 0.489 | 0.000 | 0.489 |
| 31460.50 | 0.485 | 0.000 | 0.485 |
| 31514.50 | 0.481 | 0.000 | 0.481 |
| 31526.00 | 0.477 | 0.000 | 0.477 |
| 31546.50 | 0.473 | 0.000 | 0.473 |
| 31567.50 | 0.468 | 0.000 | 0.468 |
| 31585.50 | 0.464 | 0.000 | 0.464 |
| 31614.00 | 0.460 | 0.000 | 0.460 |
| 31665.00 | 0.456 | 0.000 | 0.456 |
| 31698.00 | 0.451 | 0.000 | 0.451 |
| 31701.50 | 0.447 | 0.000 | 0.447 |
| 31728.00 | 0.443 | 0.000 | 0.443 |
| 31766.00 | 0.439 | 0.000 | 0.439 |
| 31794.50 | 0.435 | 0.000 | 0.435 |
| 31843.00 | 0.430 | 0.000 | 0.430 |
| 31888.50 | 0.426 | 0.000 | 0.426 |
| 31906.00 | 0.422 | 0.000 | 0.422 |
| 31913.50 | 0.418 | 0.000 | 0.418 |
| 31917.50 | 0.414 | 0.000 | 0.414 |
| 31921.00 | 0.409 | 0.000 | 0.409 |
| 31927.50 | 0.405 | 0.000 | 0.405 |
| 31943.00 | 0.401 | 0.000 | 0.401 |
| 31958.00 | 0.397 | 0.000 | 0.397 |
| 31985.00 | 0.392 | 0.000 | 0.392 |
| 32011.00 | 0.388 | 0.000 | 0.388 |
| 32056.50 | 0.384 | 0.000 | 0.384 |
| 32101.00 | 0.380 | 0.000 | 0.380 |
| 32107.00 | 0.376 | 0.000 | 0.376 |
| 32123.00 | 0.371 | 0.000 | 0.371 |
| 32138.00 | 0.367 | 0.000 | 0.367 |
| 32144.50 | 0.363 | 0.000 | 0.363 |
| 32149.00 | 0.354 | 0.000 | 0.354 |
| 32156.00 | 0.350 | 0.000 | 0.350 |
| 32165.00 | 0.346 | 0.000 | 0.346 |
| 32175.00 | 0.342 | 0.000 | 0.342 |
| 32185.50 | 0.338 | 0.000 | 0.338 |
| 32194.00 | 0.333 | 0.000 | 0.333 |
| 32200.50 | 0.329 | 0.000 | 0.329 |
| 32204.50 | 0.325 | 0.000 | 0.325 |
| 32215.50 | 0.321 | 0.000 | 0.321 |
| 32226.00 | 0.316 | 0.000 | 0.316 |
| 32230.00 | 0.312 | 0.000 | 0.312 |
| 32237.00 | 0.308 | 0.000 | 0.308 |
| 32243.50 | 0.304 | 0.000 | 0.304 |
| 32247.50 | 0.300 | 0.000 | 0.300 |
| 32252.00 | 0.295 | 0.000 | 0.295 |
| 32261.00 | 0.291 | 0.000 | 0.291 |
| 32269.00 | 0.287 | 0.000 | 0.287 |
| 32277.00 | 0.283 | 0.000 | 0.283 |
| 32291.50 | 0.278 | 0.000 | 0.278 |
| 32303.50 | 0.274 | 0.000 | 0.274 |
| 32308.50 | 0.270 | 0.000 | 0.270 |
| 32310.00 | 0.266 | 0.000 | 0.266 |
| 32314.00 | 0.262 | 0.000 | 0.262 |
| 32317.50 | 0.257 | 0.000 | 0.257 |
| 32324.00 | 0.253 | 0.000 | 0.253 |
| 32333.50 | 0.249 | 0.000 | 0.249 |
| 32338.50 | 0.245 | 0.000 | 0.245 |
| 32342.00 | 0.236 | 0.000 | 0.236 |
| 32346.00 | 0.228 | 0.000 | 0.228 |
| 32352.00 | 0.219 | 0.000 | 0.219 |
| 32356.50 | 0.215 | 0.000 | 0.215 |
| 32358.00 | 0.211 | 0.000 | 0.211 |
| 32360.00 | 0.207 | 0.000 | 0.207 |
| 32362.50 | 0.203 | 0.000 | 0.203 |
| 32365.00 | 0.194 | 0.000 | 0.194 |
| 32367.00 | 0.190 | 0.000 | 0.190 |
| 32370.00 | 0.186 | 0.000 | 0.186 |
| 32373.00 | 0.181 | 0.000 | 0.181 |
| 32374.50 | 0.177 | 0.000 | 0.177 |
| 32376.00 | 0.173 | 0.000 | 0.173 |
| 32381.00 | 0.165 | 0.000 | 0.165 |
| 32389.50 | 0.160 | 0.000 | 0.160 |
| 32394.50 | 0.152 | 0.000 | 0.152 |
| 32396.00 | 0.148 | 0.000 | 0.148 |
| 32398.50 | 0.143 | 0.000 | 0.143 |
| 32401.00 | 0.139 | 0.000 | 0.139 |
| 32403.50 | 0.135 | 0.000 | 0.135 |
| 32407.00 | 0.131 | 0.000 | 0.131 |
| 32410.00 | 0.127 | 0.000 | 0.127 |
| 32414.00 | 0.122 | 0.000 | 0.122 |
| 32417.50 | 0.118 | 0.000 | 0.118 |
| 32420.00 | 0.114 | 0.000 | 0.114 |
| 32424.50 | 0.105 | 0.000 | 0.105 |
| 32427.50 | 0.101 | 0.000 | 0.101 |
| 32429.00 | 0.089 | 0.000 | 0.089 |
| 32431.50 | 0.084 | 0.000 | 0.084 |
| 32434.00 | 0.080 | 0.000 | 0.080 |
| 32436.00 | 0.076 | 0.000 | 0.076 |
| 32438.00 | 0.072 | 0.000 | 0.072 |
| 32439.50 | 0.068 | 0.000 | 0.068 |
| 32444.00 | 0.063 | 0.000 | 0.063 |
| 32448.50 | 0.059 | 0.000 | 0.059 |
| 32449.50 | 0.055 | 0.000 | 0.055 |
| 32451.50 | 0.046 | 0.000 | 0.046 |
| 32457.50 | 0.042 | 0.000 | 0.042 |
| 32462.50 | 0.034 | 0.000 | 0.034 |
| 32468.00 | 0.030 | 0.000 | 0.030 |
| 32478.00 | 0.025 | 0.000 | 0.025 |
| 32505.00 | 0.021 | 0.000 | 0.021 |
| 32533.00 | 0.017 | 0.000 | 0.017 |
| 32545.50 | 0.013 | 0.000 | 0.013 |
| 32593.00 | 0.008 | 0.000 | 0.008 |
| 32634.50 | 0.004 | 0.000 | 0.004 |
| 32636.00 | 0.000 | 0.000 | 0.000 |
